# Lockdowns result in changes in human mobility which may impact the epidemiologic dynamics of SARS-CoV-2

**DOI:** 10.1038/s41598-021-86297-w

**Published:** 2021-03-26

**Authors:** Nishant Kishore, Rebecca Kahn, Pamela P. Martinez, Pablo M. De Salazar, Ayesha S. Mahmud, Caroline O. Buckee

**Affiliations:** 1 Center for Communicable Disease Dynamics, Department of Epidemiology, Harvard T.H. Chan School of Public Health, Boston, MA USA; 2 Department of Microbiology, University of Illinois at Urbana Champaign, Illinois, USA; 3 Department of Statistics, University of Illinois at Urbana Champaign, Illinois, USA; 4 Department of Demography, University of California, Berkeley, CA USA

**Keywords:** Computer modelling, Dynamic networks, Population dynamics, Infectious diseases

## Abstract

In response to the SARS-CoV-2 pandemic, unprecedented travel restrictions and stay-at-home orders were enacted around the world. Ultimately, the public’s response to announcements of lockdowns—defined as restrictions on both local movement or long distance travel—will determine how effective these kinds of interventions are. Here, we evaluate the effects of lockdowns on human mobility and simulate how these changes may affect epidemic spread by analyzing aggregated mobility data from mobile phones. We show that in 2020 following lockdown announcements but prior to their implementation, both local and long distance movement increased in multiple locations, and urban-to-rural migration was observed around the world. To examine how these behavioral responses to lockdown policies may contribute to epidemic spread, we developed a simple agent-based spatial model. Our model shows that this increased movement has the potential to increase seeding of the epidemic in less urban areas, which could undermine the goal of the lockdown in preventing disease spread. Lockdowns play a key role in reducing contacts and controlling outbreaks, but appropriate messaging surrounding their announcement and careful evaluation of changes in mobility are needed to mitigate the possible unintended consequences.

## Introduction

In response to the SARS-CoV-2 pandemic, unprecedented travel restrictions and stay-at-home orders were enacted around the world almost simultaneously. These ranged from restrictions on human movement on a local scale to travel restrictions on regional and international scales. These policies were designed to reduce the spread of the SARS-CoV-2 virus by restricting the contact between infectious and susceptible individuals and to slow the spread of the virus out of epidemic hotspots.

In general, the public’s response to announcements of lockdown policies—defined here as restrictions on local movement or long distance travel—will determine how effective these kinds of interventions are. Governments must give some warning to the public about upcoming travel restrictions to allow for necessary preparations, but a surge of travel prior to the lockdown being put in place risks the opposite of the desired effect, sending potentially infectious individuals out into previously unaffected regions around the country or internationally. In order to design effective policies in response to resurgence of SARS-CoV-2 or indeed in the context of future pandemics, understanding the human response to interventions is critical.

Analyses of aggregated data from mobile phones have been used to monitor movement patterns in the context of outbreaks^1–3^, including this pandemic^4,5^. Studies have shown that mobility patterns on local scales correlated with transmission within the city of Wuhan, for example^6,7^, and recent analyses have found associations between mobility and SARS-CoV-2 transmission in the United States^8^. Seasonal travel related to holidays, which creates a surge of travel out of cities, for example, can also have an important impact on the spread of infection^9^. Indeed, travel related to the Lunar New Year may well have spread SARS-CoV-2 across China as the epidemic started to emerge in Wuhan^10^. Since infected individuals are often infectious prior to symptoms, and many may have no symptoms at all, the possibility of infected travelers unwittingly spreading the virus during large travel movements is significant.

Here, we evaluate the effect of lockdown related travel behavior on epidemic spread by analyzing aggregated mobility data from mobile phones from multiple countries including India, France, Spain, Bangladesh, and the USA, on local and national spatial scales. We show that in numerous urban centers in the U.S. and around the world, there was a surge in travel out of cities immediately preceding lockdowns, likely in anticipation of restrictions. Importantly, this surge is not observed in surrounding areas. We observed urban-to-rural migration in each country analyzed. Drawing on this empirical evidence of the behavioral response to lockdowns, we use a simple agent-based spatial model to examine how different behavioral responses to lockdown policies could impact the spread of epidemics. We find that travel surges following announcements of lockdowns have the potential to increase movement of people to less urban areas. This movement can accelerate seeding of the epidemic in these areas compared to situations in which lockdowns do not increase travel to these regions. This change in the rate of disease exportation may therefore undermine the goal of preventing disease spread. Without detailed genetic analyses and in the absence of reliable epidemiological data in many cases, it is challenging to confirm from case data how much the changes in movement observed in the mobility data impacted the course of epidemics. However, through simulations, we show the potential for changes in behavior due to lockdown announcements to contribute to epidemic spread. This work highlights the importance of messaging and close evaluation of changes in local travel networks in the implementation of outbreak related travel restrictions.

## Results

### Pre-lockdown mobility surges and depopulation of cities

To understand the impact of lockdown announcements, which are generally made a few days before the lockdown goes into effect, we analyzed the percent change in weekday Facebook population across various urban centers in the United States, during three segments of the day: Morning, Evening, and Night. We compared these population data to a baseline of the previous 90 days conditioning on the day of the week and time of day. Daily population data, split into these three temporal bins, capture the number of people who spend the majority of that time bin in a location. Changes in population data compared to an appropriate baseline therefore capture both long-term changes in residency and short-term changes in movement into or out of that location.

**Figure 1:**
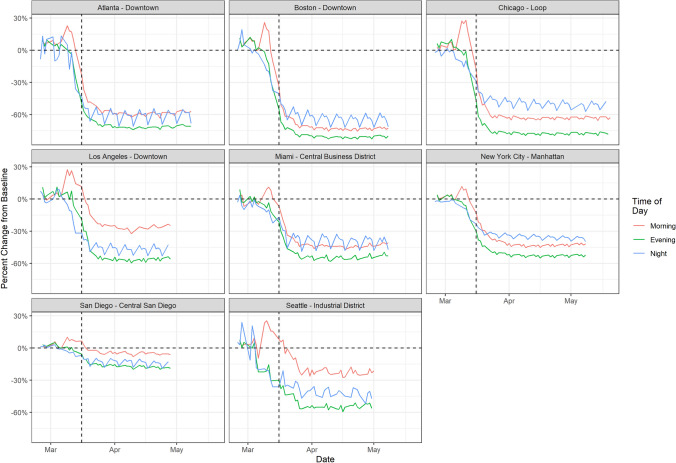
Percent change in population of weekday Facebook users in urban centers, divided into times of day. The vertical black line is March 16th, 2020, the day that the national suggested restriction on size of gatherings and non-essential travel was implemented. In all locations, there is a noted increase in the “Morning” population of Facebook users compared to the average number of Facebook users in that location during that day of the week and time of the day over the preceding 90 days.

We find that city centers around the US showed a spike in day-time mobility prior to March 16, 2020 (the day that the national suggested restriction on size of gatherings and non-essential travel was implemented), suggestive of mobility related to preparations for these restrictions (Fig. 1).This pattern was observed in urban centers across the U.S., with all locations showing a large increase in "Morning" population following the announcement of COVID-19 travel and social gathering restrictions, followed by a dramatic decline upon its implementation. This change in population data was not observed in surrounding residential neighborhoods, as shown for Manhattan in New York City compared to surrounding boroughs (Figure S1). In general, the population in most other surrounding locations stayed the same or increased following the announcement and lockdown, consistent with stay-at-home orders that would have prevented people from commuting for work. Although other factors that coincided with lockdown announcements could have induced these behavioral changes, the trends in urban mobility repeated across spatially distant cities in the U.S. suggests the common cause was likely the policies themselves (Figure S1).

**Figure 2:**
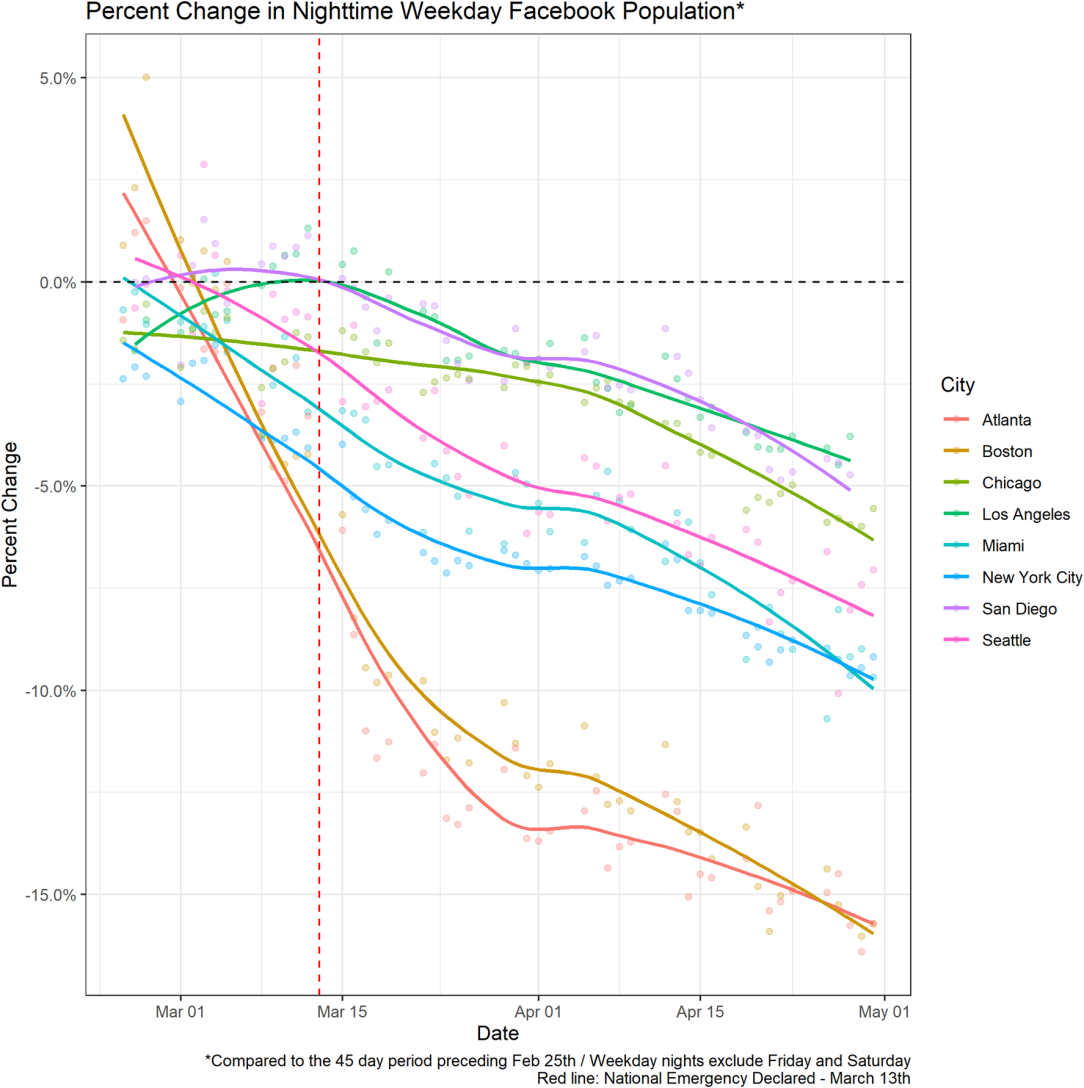
Percent change in weekday nighttime population of Facebook users by city. We can see that all cities included in the Facebook sample experience a decrease in nighttime population over the period of interest. The vertical red dotted line is when potential national COVID-19 restrictions were announced.

The data suggest that the decrease in daytime population in Manhattan was not only driven by fewer workers coming into the city during the day, but also an exodus of residents out of the city overall, characterized by a decrease in nighttime population. Note that this is unlikely to represent changes in a nighttime worker population, because we see no increases in overall nighttime population in surrounding regions S1. In Fig. 2, we show this depletion of nighttime city populations across other cities in the US, indicative of reduced residency, not just daytime activity. In New York City, the decline in residents overall was driven primarily by travel out of Manhattan, and we expect similar spatial heterogeneities may characterize population changes in other cities.

**Figure 3:**
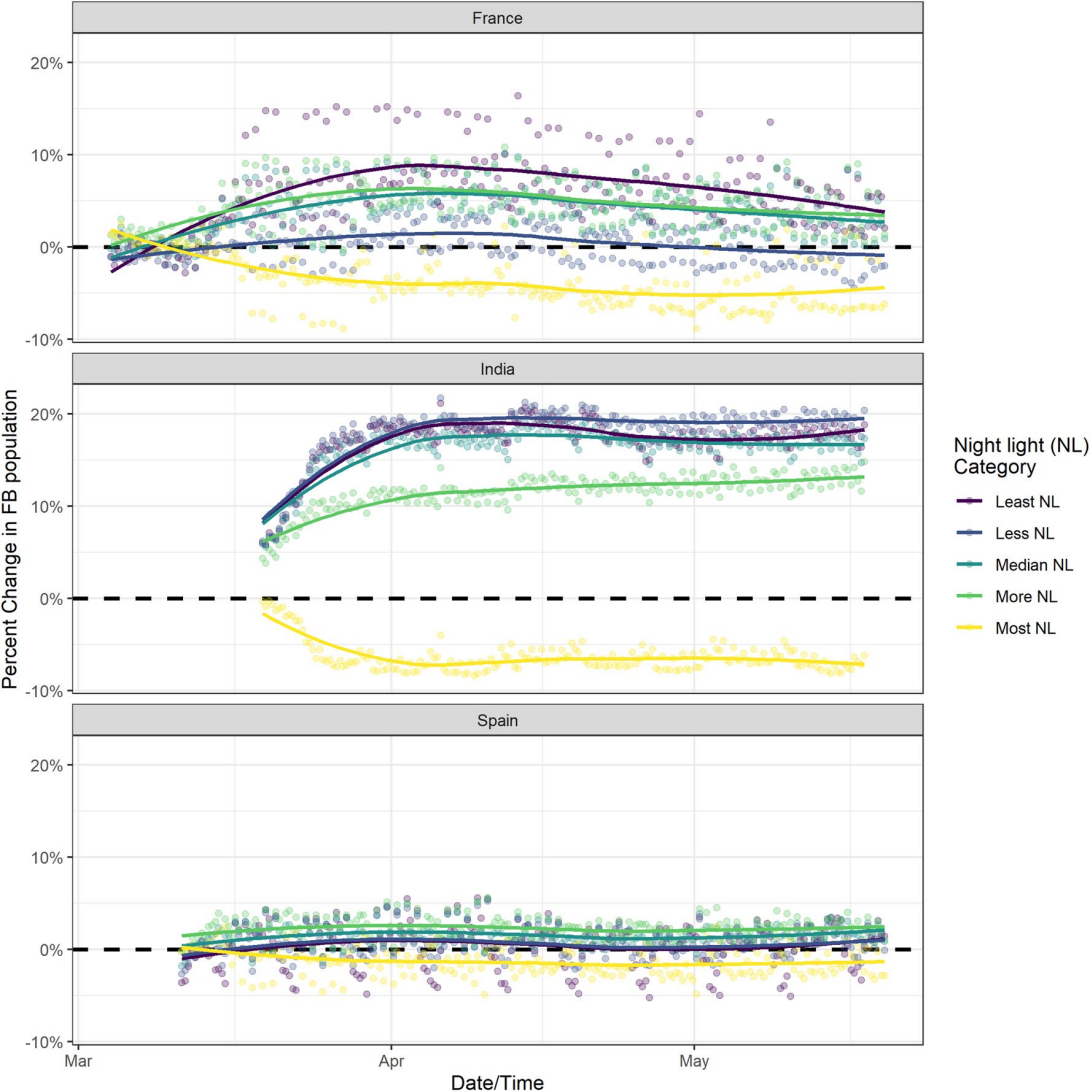
Percent change in population of Facebook users categorized by five equally sized quantiles of nightlight by country with data aggregated at the ADMIN3 level of spatial granularity.

We further evaluated changes in urban versus rural populations on a national scale in France, Spain, India, and Bangladesh; four countries for which Facebook data pipelines were available to cover the timing of lockdowns. In Fig. 3 regions in these countries are divided into five equally sized quantiles of nightlight ($\frac{{\text {nW}}}{{\text {cm}}^2sr}$)^11^, which correspond to population density and reflect the urban-to-rural gradient. In each country, to varying degrees, there was a consistent decrease in population in areas with the highest nightlight intensity (urban centers) and a reciprocal increase in population in less electrified regions (more rural areas).

**Figure 4:**
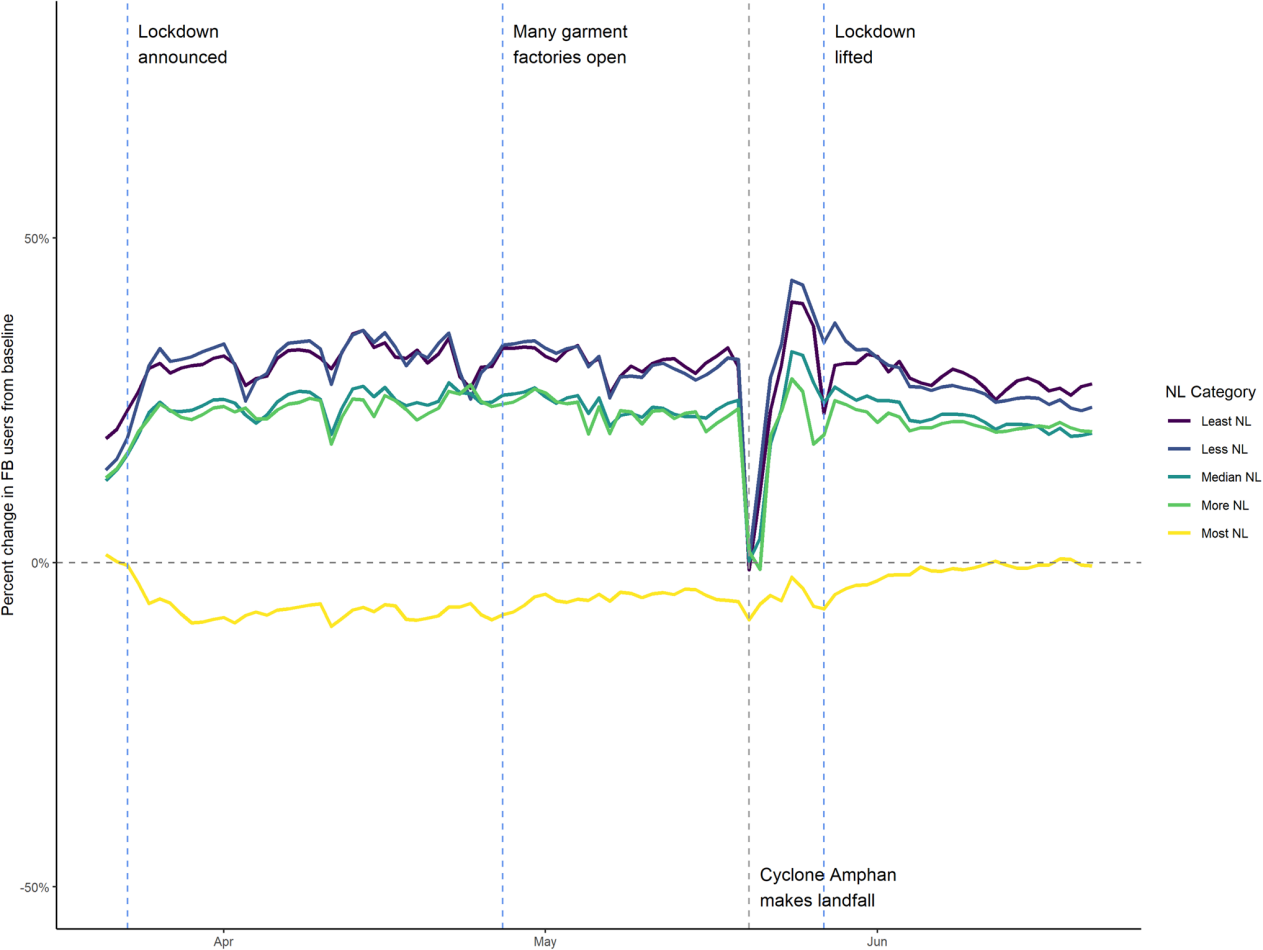
Percent change in population of Facebook users categorized by five equally sized quantiles of nightlight in Bangladesh with data aggregated at the ADMIN2 level of spatial granularity.

In Bangladesh, we find a substantial decline in population in areas with the highest nightlight intensity—primarily in the capital, Dhaka, and areas with a high concentration of garment factories. The announcement of the lockdown in early March, and the closing of the garments industry, was followed by large movements of people from these densely populated urban areas to more rural areas^12–14^. Figure 4 shows the striking pattern of population decline in urban areas in March, followed by a gradual increase as the garments industry and other workplaces opened in late April. We are unable to use Bangladesh data for movement analysis or compare it directly to data from India, France and Spain due to differences in the spatial granularity of Facebook data.

### Simulating travel behavior in a metapopulation model of SARS-CoV-2

To examine the potential epidemiological implications of these behavioral responses to lockdowns, we implemented a metapopulation model reflecting the general behaviors we measured in the Facebook data. As shown in Figure S2, which depicts results from an epidemic without a lockdown or other interventions, we initiated the epidemic in an “urban” center (identified by a black outline) with a higher population density and evaluated the epidemic spread across all other “non-urban” areas, with travel determined by a gravity model of movement. We then varied travel and infection dynamics based on timing in relation to lockdown announcements and implementations (Figure S3).

We evaluated the probability of travel ($\alpha _0$) under varying parameter values in the null model (i.e. no change in movement due to lockdown) with the goal of simulating a depopulation of the location that served as the urban center that was similar to the empirical data shown in Fig. 2. Across a variety of scenarios, an $\alpha _0$ of 0.01 (baseline daily travel probability) resulted in an at least 10% decrease in the population size of the urban center over the course of 60 days (Figures S4, S5, S6).

### Pre-lockdown travel surges lead to faster and further initial spread of the simulated epidemic

In our simulations, lockdowns affect behavior in two ways, reflecting the trends observed from the Facebook data in many locations: first, between announcement and lockdown implementation, contact rates within populations ($\beta _1$) temporarily increase due to activities undertaken to prepare for the lockdown, and subsequently decrease once the lockdown takes effect ($\beta _2$). Second, travel from urban to less urban locations also changes prior to ($\alpha _1$) and following ($\alpha _2$) lockdown. We evaluated each possible parameter combination against a relative baseline where there is no travel surge and no increased contact rate during the period between lockdown announcement and implementation.

**Figure 5:**
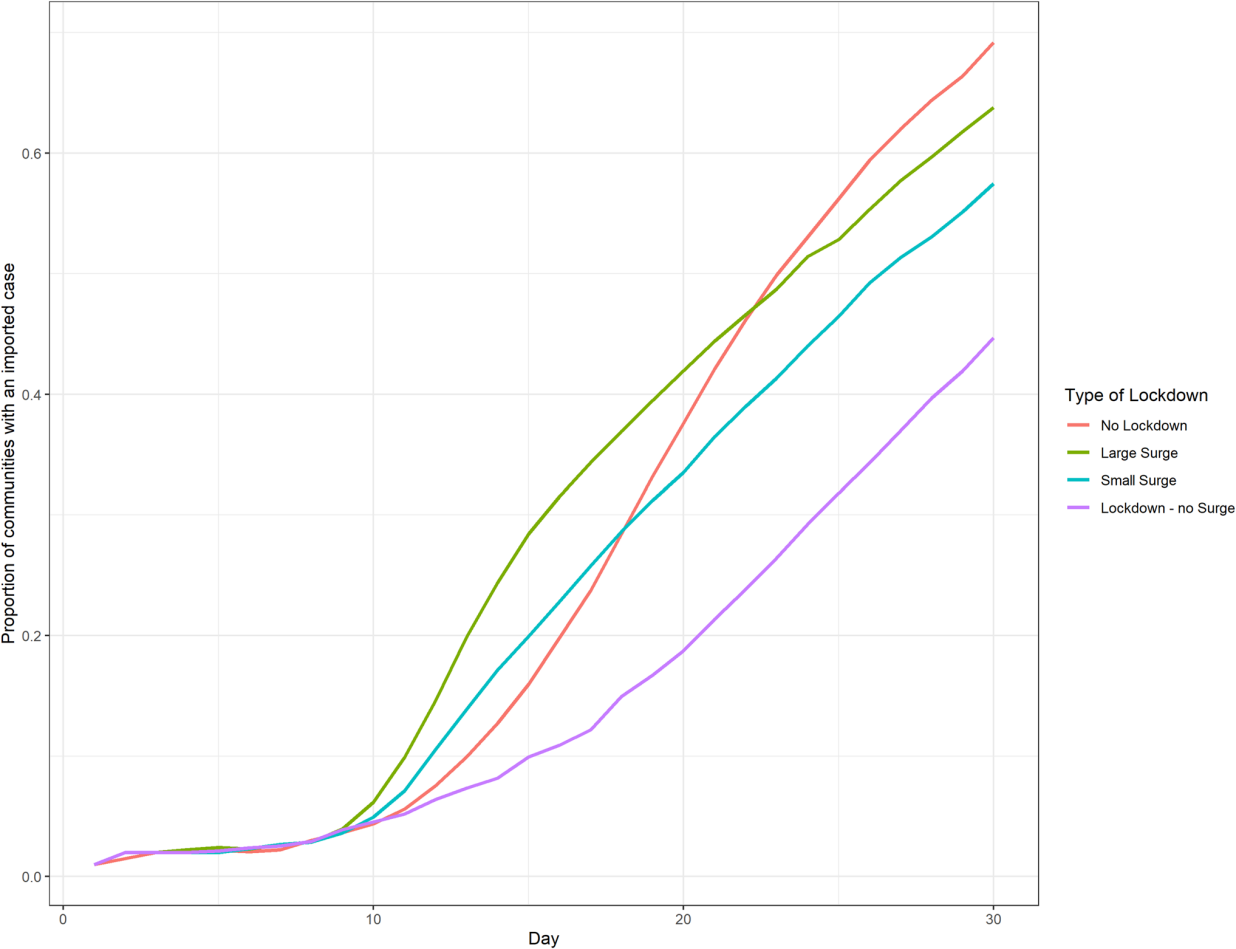
Average proportion of simulated communities with an imported case. Initially the epidemic spread quicker in simulations with a large or small surge, however, simulations with no lockdown result in a larger overall epidemic size and eventually spread more rapidly.

**Figure 6:**
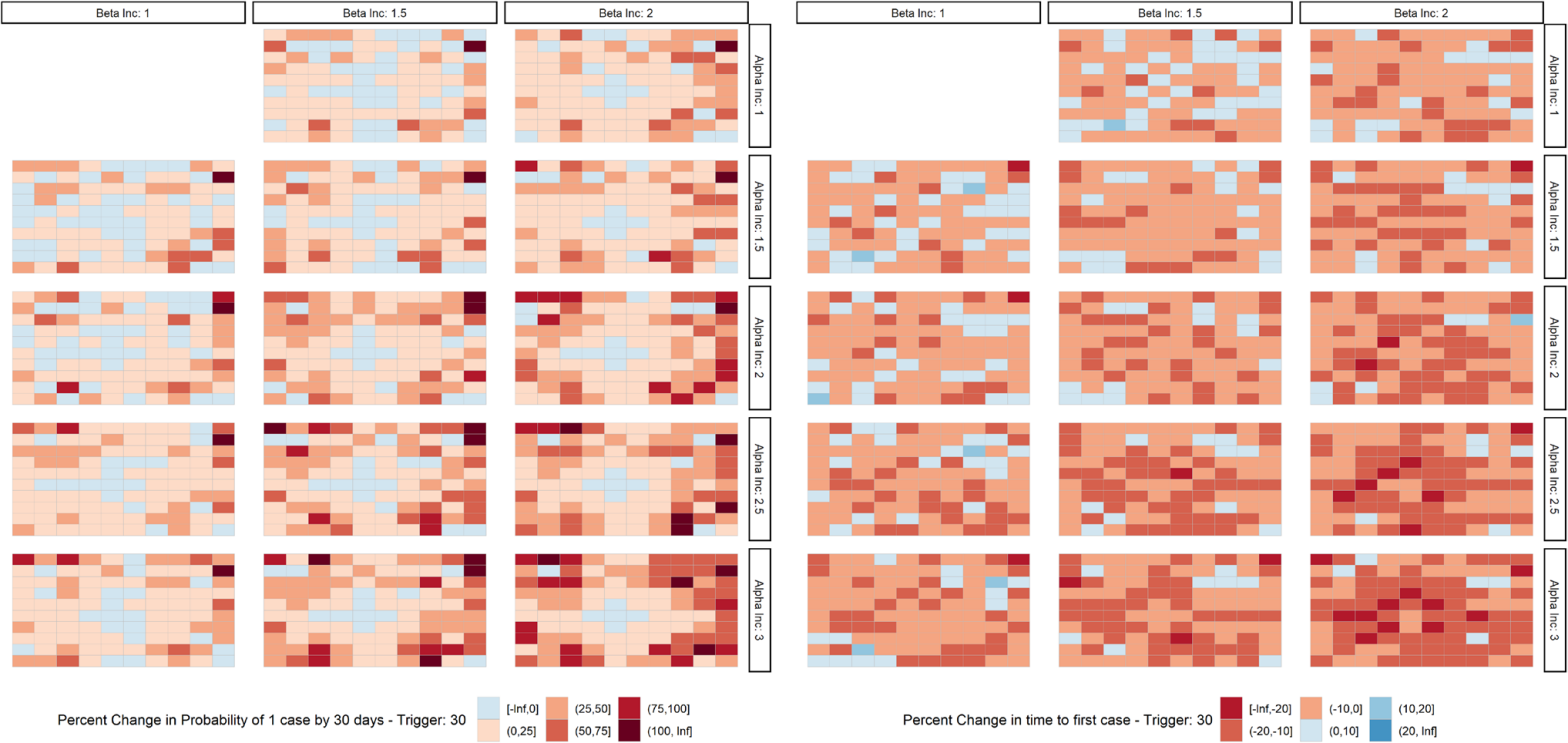
Percent change in probability of having at least 1 case by 30 days (left); Percent change in the number of days till the first case (right).

While lockdowns can decrease the spread of the epidemic if they are maintained effectively over time, our simulations show that travel surges at the beginning of an epidemic can lead to increased exportation of cases out of the epicenter (Fig. 5). In fact, these results show that travel surges have the potential to initially spread the disease faster than if no lockdown at all had been implemented. In our simulations, changes in contact rates ($\beta _1$), reflecting the changes in local movement observed in the Facebook data, and travel ($\alpha _1$), reflecting the changes in long distance movement, following the announcement of a lockdown cause this increased rate of exportation of disease, with the former contributing more than the latter; however, there is a clear multiplicative effect as seen in Fig. 6. Figure 6 (left) describes the relative probability of having an outbreak in a region within the first 30 days of the simulation, compared to a scenario where there is no change in $\alpha$ and $\beta$ during the $L_1$ period. This highlights the overall risk that these simulated communities face over the course of the epidemic, as well as the speed of outbreak spread.

Given the novel nature of SARS-CoV-2 we have defined the detection of a single case in a given location as a clear metric of the potential for initial seeding of an epidemic. Figure 6 (right) evaluates the percent change in the number of days until an outbreak occurs, compared to the baseline scenario. This demonstrates the relative speed with which an epidemic is able to reach surrounding communities. As contact rates and travel increase, there is a corresponding increase in seeding of epidemics in new locations, as well as faster spread to all locations. This occurs because an increase in $\beta _1$ results in a larger number of local cases available for travel while an increase in $\alpha _1$ results in an increased overall probability of those cases traveling.

**Figure 7:**
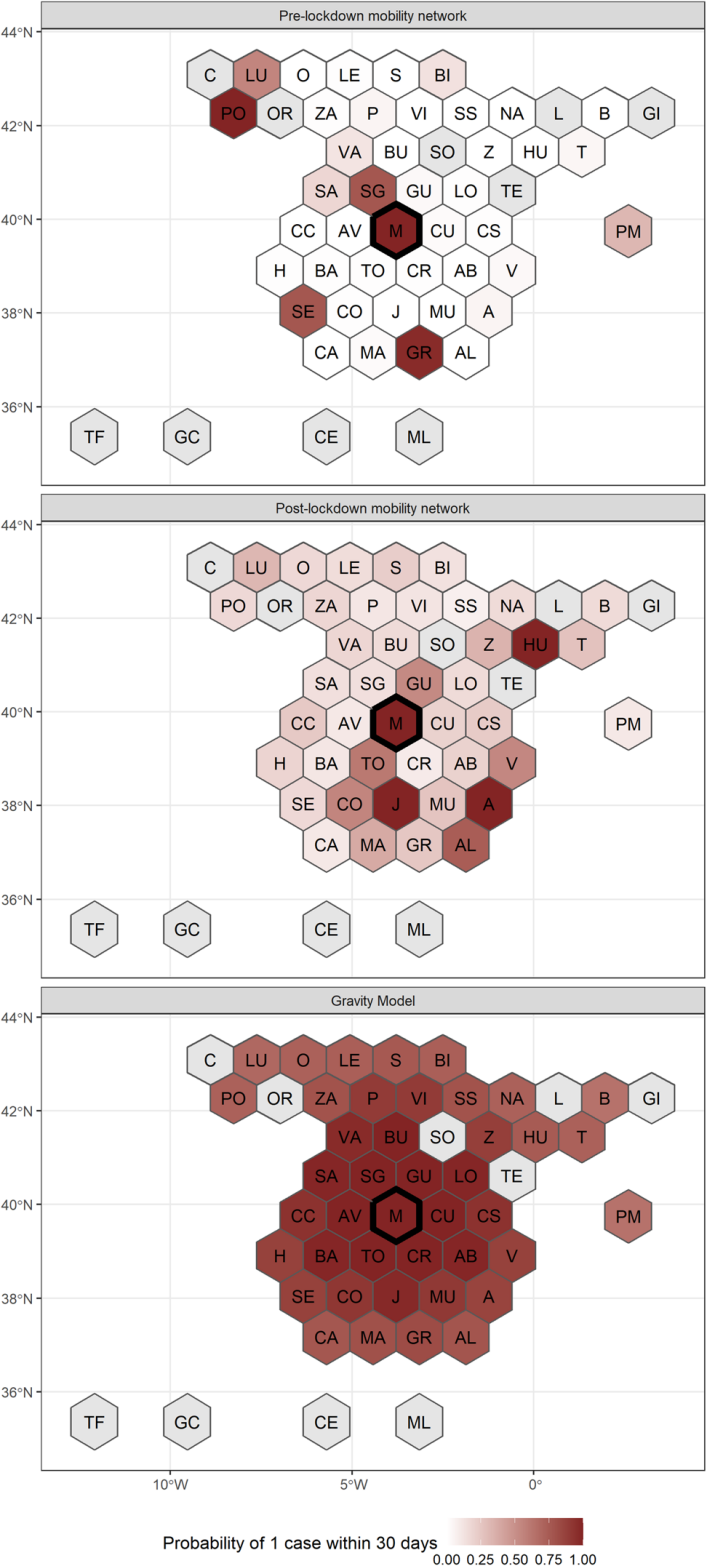
Simulated probability of epidemic within 30 days in provinces of Spain comparing clustered post-lockdown mobility matrices to pre-lockdown ones generated from Facebook movement data as well as a simple gravity model.

In extended analyses using daily mobility matrices from Spain we identified similar phenomena as in our simulation models. Adapting the simulation models to integrate mobility matrices between provinces in post-lockdown Spain (see “Methods”), we compared epidemic spread under (1) “normal” travel behavior, assuming a continuation of observed baseline travel, (2) observed travel surge behavior following the lockdown announcement, and (3) a null model where mobility was calculated using a gravity model. Strikingly different epidemic patterns emerged from these models (Fig. 7). “Normal” travel behavior led to the rapid dissemination of the epidemic due to long-range movements between travel hubs (Fig. 7A), whereas the observed travel surge following the lockdown announcement led to the spread of SARS-CoV-2 in less urban areas around the epicenter and widespread dissemination of the virus (Fig. 7B). Both of these data-informed models were in stark contrast to the gravity model. All three models assumed similar probabilities of overall travel as determined by the epidemic parameters and only vary the travel networks themselves.

While we do not expect a simple simulation model to exactly replicate complex epidemic patterns, qualitatively we do see similar spatial dynamics as were observed during pre- and post-lockdown periods. The pre-lockdown mobility network mimics real-world exportation of cases to regions that are distant but highly connected to Madrid such as Pontevedra (PO). The post-lockdown mobility network, on the other hand, simulates the spread of the epidemic regionally, but still allows for identification of potentially high risk regions not identified in a simple gravity model.

### Rapid implementation of lockdowns after announcement can decrease the exportation of cases

The choice of timing between lockdown announcement and implementation must balance the increased risk of exportation from longer delays with the need to provide enough warning for people to adequately prepare for the lockdown. Our model shows that decreasing the time between announcement and lockdown implementation reduces the number of exported cases. As shown in Figure S7, an $L_1$ period of 0 days resulted in no discernible increase in risk of an epidemic across all locations compared to the baseline. However, as we increased $L_1$, the probability of having at least one case by thirty days increased in most non-urban locations. This effect was especially notable in locations far removed from the urban center. Importantly, the speed of the exportation of the epidemic was driven by both the duration of the $L_1$ period and modification of the travel surge as defined by $\alpha _1$ and $\beta _1$. With an $L_1$ of 7 days, an $\alpha _{inc}$ of three and a $\beta _{inc}$ of two, it is the locations that are closest to the urban center that have an exceptional decrease in the average number of days until the first case.

## Discussion

The COVID-19 pandemic led to an unprecedented and nearly simultaneous set of lockdown policies that were implemented globally. The extent to which they were effective in containing the spread of SARS-CoV-2 remains unclear, but the impact of physical distancing policies on human mobility is measurable at scale for the first time due to the widespread availability of data from mobile phones. Here we have shown that characteristic travel behaviors were repeated across different cities and countries around the world in response to lockdowns, with pre-lockdown surges in local activity and rapid urban-to-rural migration characterizing the human response to the policies. Our results suggest that these social responses are predictable and generalizable to some extent, and must be taken into account when planning the implementation of future lockdown policies, particularly with respect to messaging and surveillance.

The mobility data analyzed here highlight that even with lockdown orders, barring draconian policies, populations do not stop moving completely, but rather change their mobility patterns (Figure S8). Our simulations, informed by these empirical data, show that both travel and local contact rates can play a key role in the increased risk of exportation of cases to non-urban locations following announcement of a lockdown. The outcomes of our model indicate that a temporary increase in local contact rates and mobility results in more epidemic seeding in less urban areas compared to if the lockdown were implemented without these increases (Fig. 5). Importantly, long-distance urban-to-rural travel drives the speed of epidemic spread and greatly reduces the time until the first case, particularly in locations close to the urban center. This effect is modulated by the duration of the time between announcement and implementation, with longer time until implementation resulting in increased probability of having an epidemic and decreased average time until the first case. Understanding these patterns will be key in the design and implementation of future lockdown policies.

There is some evidence that the long-distance seeding of SARS-CoV-2 cases due to travel behavior may have occurred. For example, an analysis of the genetic relatedness of viral genomes found that many outbreaks across the United States were seeded by travelers from New York City^15^, and an analysis of SARS-CoV-2 viral genomies in Bangladesh found movement was a key driver of disease spread^16^. Case data from Spain^17^ also show increases in cases across a wide range of locations following the lockdown implementation. While many factors likely contributed to the similarities in the epidemic curves across locations, the increase in travel in the mobility data suggests seeding from urban areas may have played a role. Given the limitations of of epidemiological case data, more detailed genomics analyses and reconstruction of transmission networks are necessary to disentangle the contributions of these many factors.

Many simplifying assumptions were made in the simulation model for clarity, including homogeneous mixing within locations on the lattice, a gravity model for connectivity, and the inclusion of a single urban center. Additionally, we assumed transmission dynamics were the same between symptomatic and asymptomatic individuals, and that symptomatic individuals stopped traveling immediately. We further assumed that increases in movement observed in the data following lockdown announcements were a reasonable proxy for increased contact rates, particularly in light of the anecdotal evidence of “panic buying” However, in future outbreaks, interventions such as masks and social distancing, which were not consistently implemented in many places when lockdowns were first initiated, may reduce the meaning of this proxy. Finally, the mobility analyses absorb the limitations of the Facebook data, which are limited to Facebook users with location services enabled. These users are just a subset of the population, and may represent a biased sample. However, the consistency of the patterns across countries suggests that the data are indeed capturing a real phenomenon. Despite these limitations, our results highlight the need for careful implementation of lockdowns to mitigate their potential unintended consequences.

Strategies for mitigating travel surges will greatly depend on the reasons behind people’s movement. Movement of people to rural homes from urban centers due to sudden lack of work from the pandemic^12^ will require different interventions and messaging than people choosing to leave crowded cities for more remote second homes^18^. For example, depending on the setting, lockdown announcements could include messaging on how to safely prepare and expectations of the local supply chain to decrease instances of panic buying and hoarding, thereby decreasing the spike in local travel immediately preceding a lockdown^19^, and governments could consider providing resources needed for people to stay. Decreasing the window of time between announcement and implementation of lockdown policies could also reduce both the local contact rate and the probability of out migration while balancing the needs of the population. While the exact policy implications will be context specific, our results suggest that lockdown announcements should be accompanied by additional messaging and resources to minimize potential unintended consequences. In particular, there are longstanding, global disparities in access to healthcare between urban and rural areas^20,21^, which have been further exacerbated by the SARS-CoV-2 pandemic^22^. Travel surges of the kind we observed here thus necessitate increased coordination, surveillance, testing, and treatment in rural areas that historically are understaffed and under resourced^23^.

## Methods

### Mobility data

Facebook’s Data for Good team developed and provides access to the Geoinsights portal to provide movement and population level data in response to crises^24^. This interface allows researchers and response workers to request aggregated and anonymized datasets generated by an open cohort of individuals who are: (1) Facebook users; (2) have a smartphone, and; (3) are providing information through the Facebook app by having location services enabled. Data are requested for a geospatial region and defined by a spatial bounding box. For this analysis we used the movement and population datasets.

When the data aggregation pipeline is initiated, all individuals who are in the cohort described above and inside the bounding box contribute information to the datasets. For each user, location information is collected, and user location is categorized to Bing Tiles. The resolution of the Bing Tiles used varies by type of dataset with population data being offered at a higher resolution than movement data due to computational restrictions. Data are then aggregated into 8-h bins. Population is determined by the modal location for each individual during this 8-h bin. Movement for a given 8-h bin is defined as a vector of transition with the destination being the modal location in the current 8-h bin and the origin being the modal local for the preceding 8-h bin. For each population tile and movement vector, Facebook provides a baseline which is calculated as the average number of users who were categorized as being in a given location (population) or who had made a given directional transition (movement) during the baseline period, conditional on day of week and time of day. The baseline period is defined as the 45-day period preceding the initiation of the pipeline for movement data and the 90-day period preceding the initiation of the pipeline for the population data.

### Selection of data sources

On February 27th, Facebook’s Data for Good team initiated the data collection pipeline for major cities in the United States of America. In the following weeks bounding boxes, and subsequent pipelines, were generated for regions as requested, including internationally. Our analyses are constrained to the locations with available data for the relevant time periods, and we use a combination of Facebook mobility data and nightlight data in different areas, as described below.

We analyzed the percent change in weekday Facebook population during various segments of the day compared to a baseline of the previous 90 days, conditioning on the day of the week and time of day in various urban centers in the United States. We use this measure of percent change as it allows for us to standardize population change against an expectation and therefore account for within-region fluctuations in weekly and daily population. Large deviations from baseline (or a percent change close to 0%) are not expected in the short term without some exogenous event.

We performed sub-city level analyses for several U.S. cities for which we had contemporaneous Facebook mobility data. We restricted our regional sub-city analysis to New York City (S1) as (1) there are clear geographic borders (boroughs) with heterogeneity in the demographics of the population and land use in each region, (2) there were a large number of users included in the Facebook data set for each region, and; (3) the boroughs are of a large enough spatial scale to allow Facebook to capture highly granular movement and population data. City level analyses were restricted to the United States as Facebook initiated a city specific data collection pipeline for select cities on February 27th, well before the implementation of lockdown measures. Country level analyses were restricted to Spain, India, and France as all three countries quickly implemented strict lockdown measures, and Facebook initiated data collection pipelines for the whole country before these measures were put into place.

### Model initialization

To assess the potential impact of different lockdown implementations and travel restrictions, we developed a simple metapopulation model, consisting of 100 communities, evenly spaced on a ten-by-ten lattice. One community in the center represents an “urban” area with a higher population size and population density than the other 99 “non-urban” locations. We make the simplifying assumption that all non-urban locations are homogeneous in terms of size and density and only differ in their distance from the urban. We seed an epidemic in the urban center with five initial cases. Within each community, the epidemic follows a density-dependent stochastic Susceptible-Exposed-Infectious(Asymptomatic)-Infectious(Symptomatic)-Recovered natural history. At each time step, all susceptible individuals have a chance of infection from the infectious individuals in their community, based on the parameter beta (i.e., force of infection). Asymptomatic and symptomatic cases are assumed to have the same beta, meaning the only difference between them in the model is whether or not they show symptoms. Detailed parameters of the outbreak are listed in Table 1. Individuals that are symptomatic (I) or asymptomatic (A) proceed through their disease history and approximately 10% of each compartment are removed into the recovered (R) compartment each time step for an average recovery period of 10 days^25^.

**Table 1:** Simulation parameters.

Parameter	Value
Number of communities	100
Size of urban center	4000
Size or “non-urban” areas	2500
Area of urban center	4
Area of “non-urban” areas	10
Number of initial infections	5
Latent period	5 days
Infectious period	10 days
Proportion symptomatic	0.5
$\alpha _0$ (travel)	0.01
$\alpha _{inc}$	1, 1.5, 2, 2.5, 3
$\alpha _{dec}$	0.5, 1
$\beta$ (force of infection)	0.0015
$\beta _{inc}$	1, 1.5, 2
$\beta _{dec}$	0.5, 1
$\omega$ (days between announcement and lockdown)	0, 3, 7
$\delta$ (cases to trigger lockdown)	10, 30
Time steps	60 days

Following the time step specific movement through the disease generation process individuals in each community are given a chance to travel. This travel is driven by three factors: (1) the probability that an individual travels out of a given community, $\alpha _0$; (2) the probability that an individual from community i travels to community j, given that they will travel out of community i, $p_{ij}\mid \alpha _0$ and; (3) the disease status of the individual. All individuals that are in the S, E, A and R compartments are able to travel. Here we assume that individuals who are symptomatic and infectious will self-isolate and not travel. We first calculate the number of individuals that leave each compartment in each community, and then distribute them into the same compartment in another community, depending on the probabilities described above. As seen in Figs. 2 and 3, we see wide ranging levels of depopulation in urban areas. In the most acute cases, such as in urban centers in Fig. 1, we see an approximate 40% decrease in the nighttime population. However, in country level analyses this can vary significantly. We have tuned the $\alpha _0$ parameter in our model to result in an approximately 10% reduction in our “urban” population over the length of our model run. The value $p_{ij}\mid \alpha _0 = \frac{M_{ij}\alpha _0}{\sum _1^jM_{ij}\mid \alpha _0}$ where $M_{ij}$ is the i specific normalized value of a simple gravity model defined as:

$$\begin{aligned} M_{ij}\mid \alpha _0 = \frac{pop_i \times pop_j}{(\mid row_i - row_j \mid + \mid col_i - col_j \mid )^2} \end{aligned}$$

Here the values for row and col return the row and column number of the community in our ten-by-ten lattice. Given that an individual moves, the location that they move to is determined by a gravity model with locations that are closer and locations which are more heavily populated (i.e. the urban center) receiving a higher probability of travel.

### Incorporating mobility data directly into the simulation

Using the movement between tiles data from Facebook, we calculated the time-varying number of transitions between all provinces of Spain. We then constructed a mobility matrix for transitions from location *i* to location *j*, standardized for all travel out of location *i* for that day. This resulted in a value bounded between 0 and 1 describing the probability of travel to location *j* from location *i* given that an individual traveled out of location *i* on that given day. In the Facebook mobility data from Spain, we had 5 days of pre-lockdown data. For our simulation model we generated three time-varying mobility matrices: (1) the first which followed the true change in mobility network; (2) the second which randomly sampled from the mobility network of the pre-lockdown days through the entire period of the simulation, and; (3) the first which simulated a simple distance based gravity model with no input from the mobility data. We also expanded the initial pre-lockdown period to 14 days to allow for an initiated epidemic to propagate in our simulation by randomly sampling from the pre-lockdown mobility matrices.

### Timing and tuned parameters

We designed our model to describe three distinct periods of time: (1) $L_0$, the period before any lockdown measures are announced or implemented, (2) $L_1$, the period of time after announcement of lockdown, but before implementation; and (3) $L_2$, the period of time after the implementation of the lockdown (Figure S3). As described above, the initial parameters of the disease generation process and movement were controlled with $\alpha _0$ and $\beta _0$, which were tuned empirically. We varied six parameters which influenced these initial parameters to evaluate the impact of differential implementation of lockdowns as shows in Figure S3.

- $\alpha _{inc}$: A multiplicative factor which describes the increase in $\alpha _0$ during the $L_1$ period resulting in $\alpha _1$. We used this variable to simulate the increase in movement out of urban areas. $\alpha _{inc}$ is assumed to be constant throughout the $L_1$ period.
- $\alpha _{dec}$: A multiplicative factor which describes the decrease in $\alpha _0$ during the $L_2$ period resulting in $\alpha _2$. We used this variable to simulate the reduction in movement between all locations resulting from the implementation of a lockdown.
- $\beta _{inc}$: A multiplicative factor which describes the increase in $\beta _0$ during the $L_1$ period resulting in $\beta _1$. We used this variable to increase the force of infection in areas where an epidemic had already started to simulate the increase in the contact rate between individuals due to greater local movement.
- $\beta _{dec}$: A multiplicative factor which describes the decrease in $\beta _0$ during the $L_2$ period resulting in $\beta _2$. We used this variable to decrease the force of infection in the areas where an epidemic had already started to simulate the decrease in the contact rate likely after the implementation of lockdown measures.
- $\delta$: The number of local symptomatic cases necessary for announcement and implementation of lockdown measures. Here we assumed that all symptomatic cases were immediately identified.
- $\omega$: The amount of time between announcement of a lockdown and implementation.

### Metrics

We simulated the stochastic epidemic 100 times. In each of the 100 communities, we calculated the proportion of simulations in which that community had at least one case by day 30. We also calculated the average time to first infection across simulations in each community. We compared these two metrics across variations of the six parameters described above. For our primary analysis we held $\delta$ constant as it did not directly affect our question of interest. We subsequently varied $\delta$ to evaluate the sensitivity of our model.

## Supplementary Information

Supplementary Information.
