# Supplementary material for: Lockdowns result in changes in human mobility which may impact the epidemiologic dynamics of SARS-CoV-2
Source: Sci Rep. 2021 Mar 26;11:6995. doi: 10.1038/s41598-021-86297-w (PMC7997886; doi:10.1038/s41598-021-86297-w)
Supplement: Supplementary file 1 — Supplementary Information. [file 41598_2021_86297_MOESM1_ESM.pdf]

Supplementary Materials

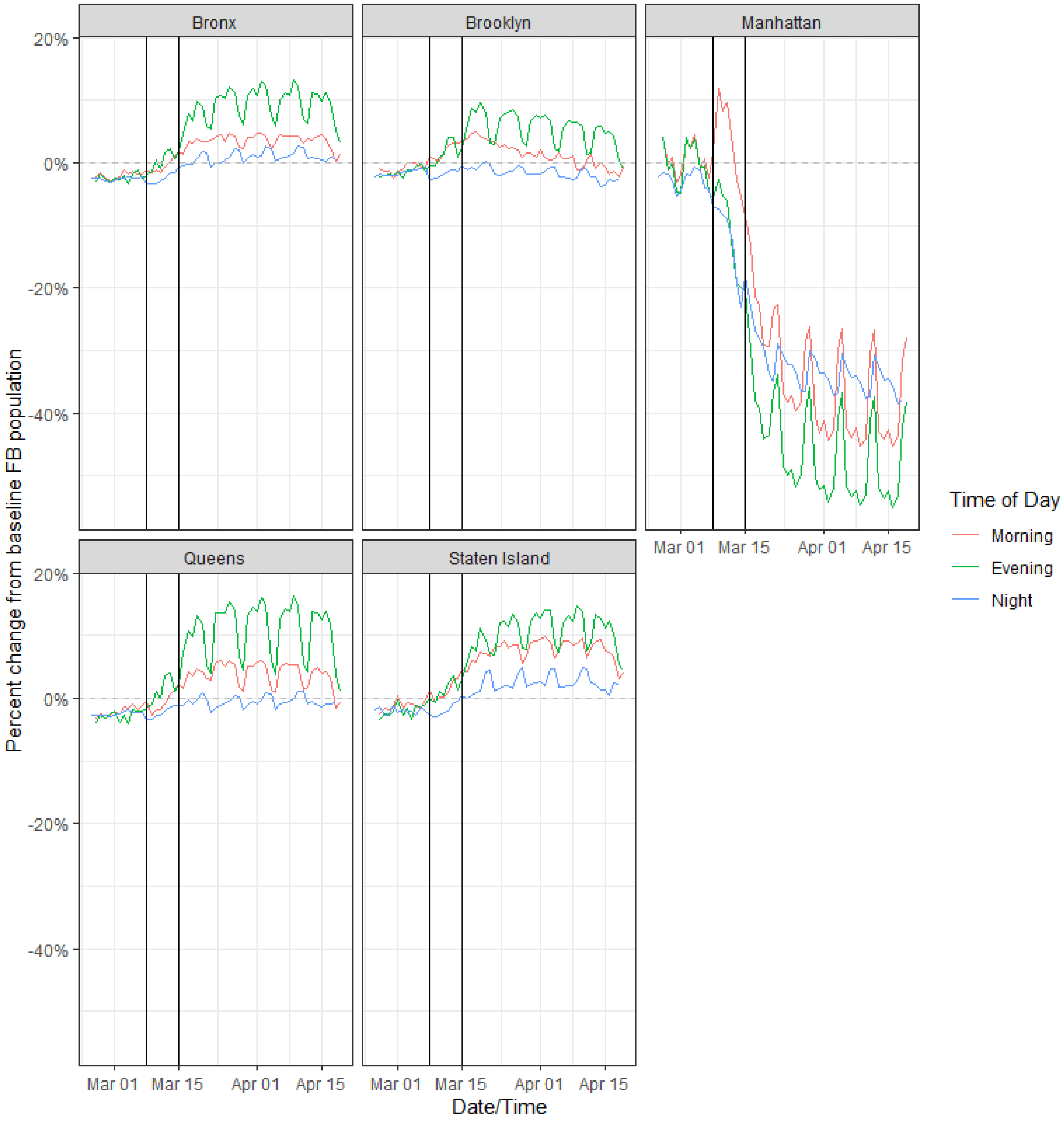

Figure S1. Percent change in population by time of day in New York City. Percent change is calculated against a baseline for the same region defined as the average population in a given region over the 90 days preceding the first day of data collection (a static period) conditional on time of day and day of week. This standardized measure allows for comparison between regions which may have large variability in weekly and daily populations. By comparing to surrounding regions (in this case boroughs) we find that the morning population in Manhattan spiked in the days preceding a travel restriction implementation and then decreased steadily afterwards. This same spike and subsequent decrease in population is not expected or observed in surrounding regions which are more residential than Manhattan.

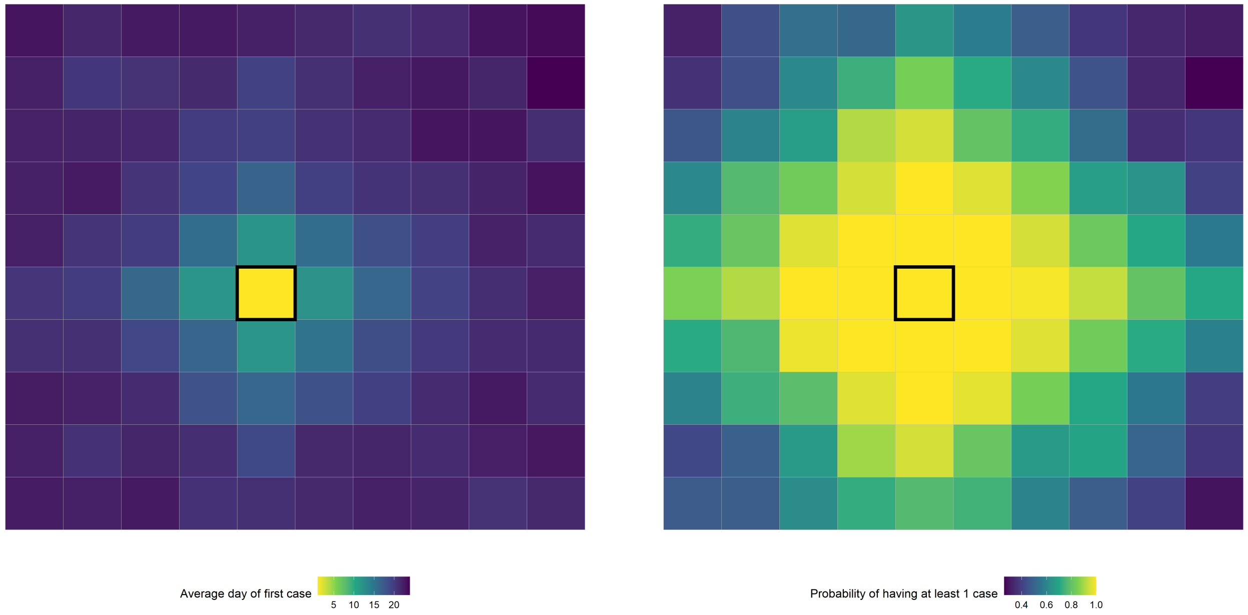

Figure S2. Results of a simulation evaluating: probability of at least one case by 30 days (right) and the average day that the first case appeared (left). The location outlined in black is the "urban" center with a larger population size and density. All other locations are "non-urban" with the same population size and density.

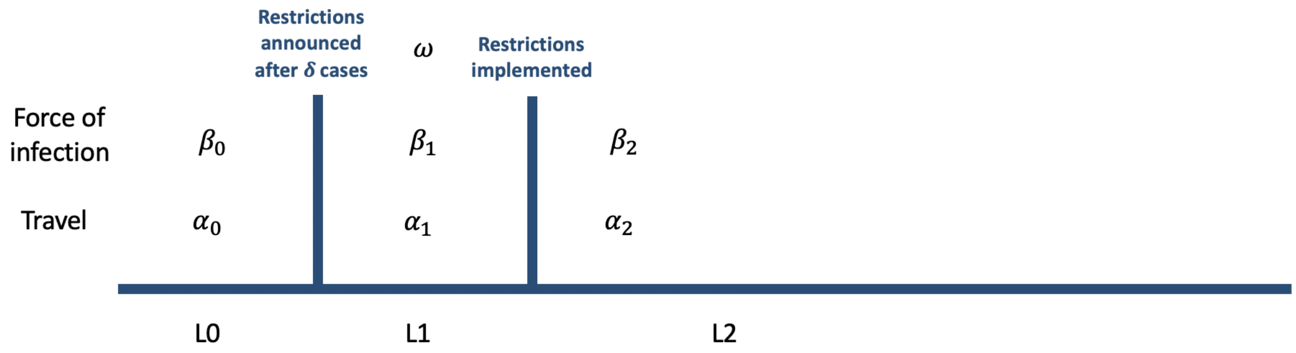

Figure S3. Diagram of the lockdown process and the values used for disease generation in communities and movement between communities.

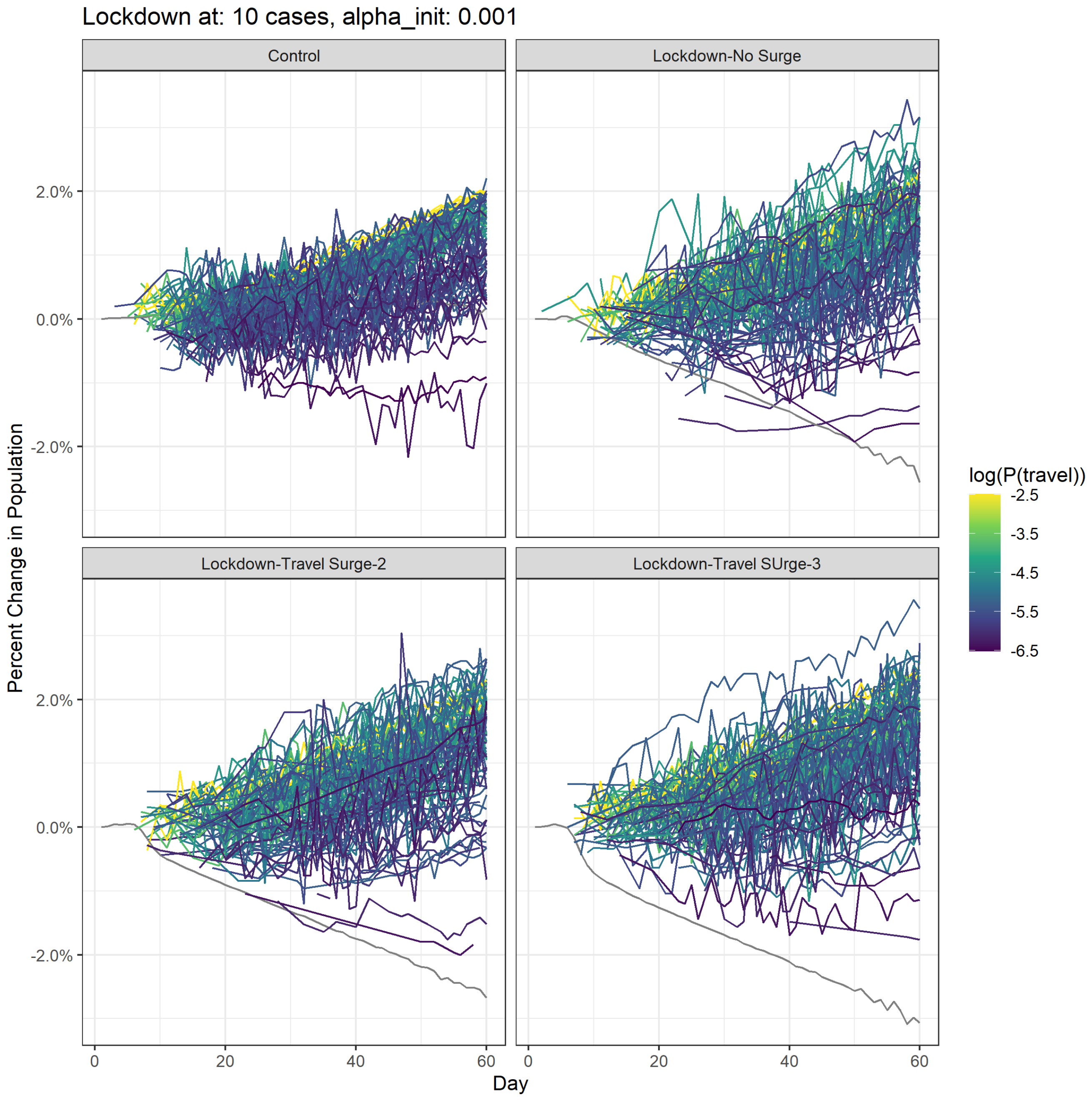

Figure S4. Depopulation of urban center with α_0 of 0.001.

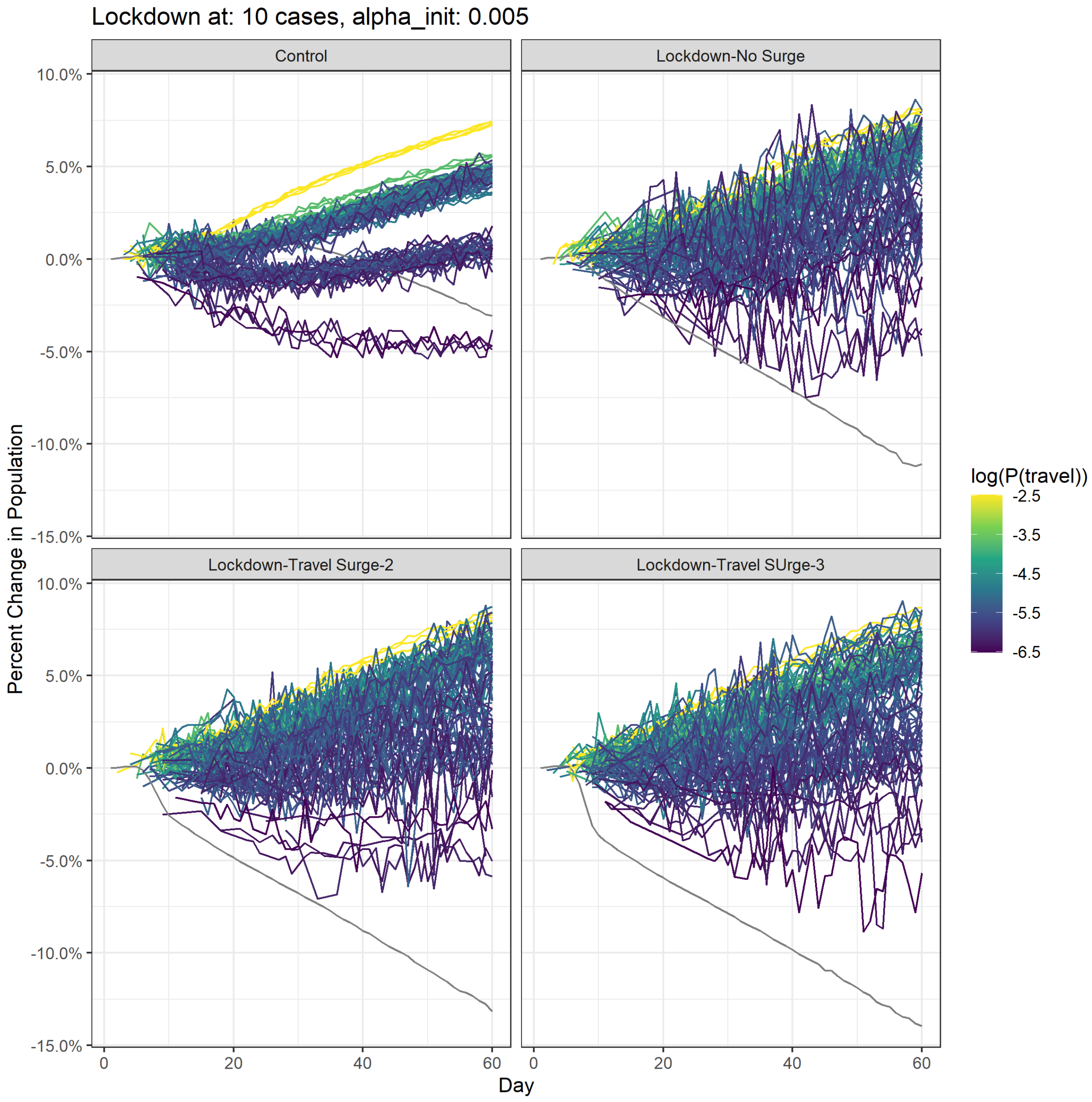

Figure S5. Depopulation of urban center with α_0 of 0.005.

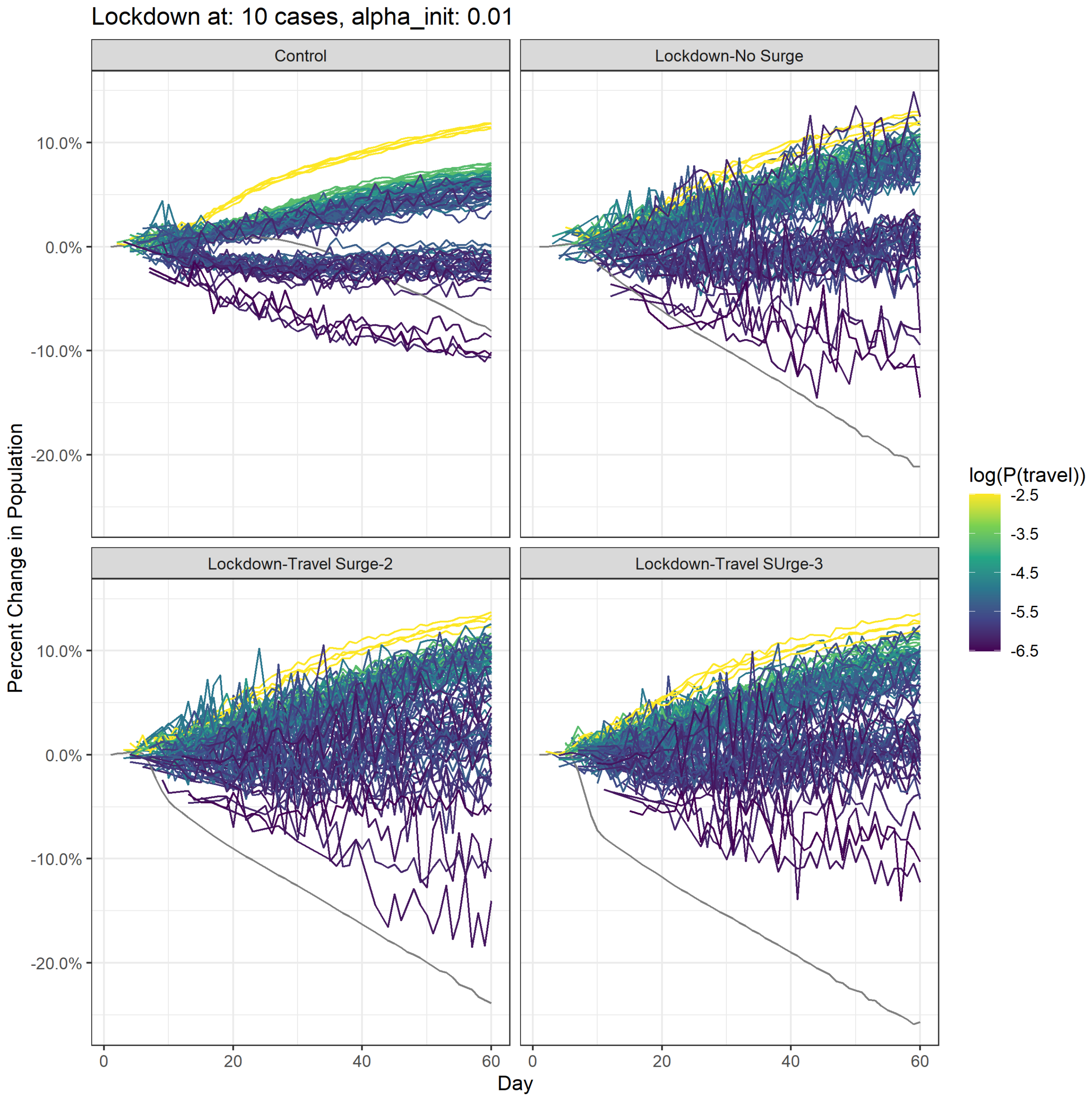

Figure S6. Depopulation of urban center with α_0 of 0.01.

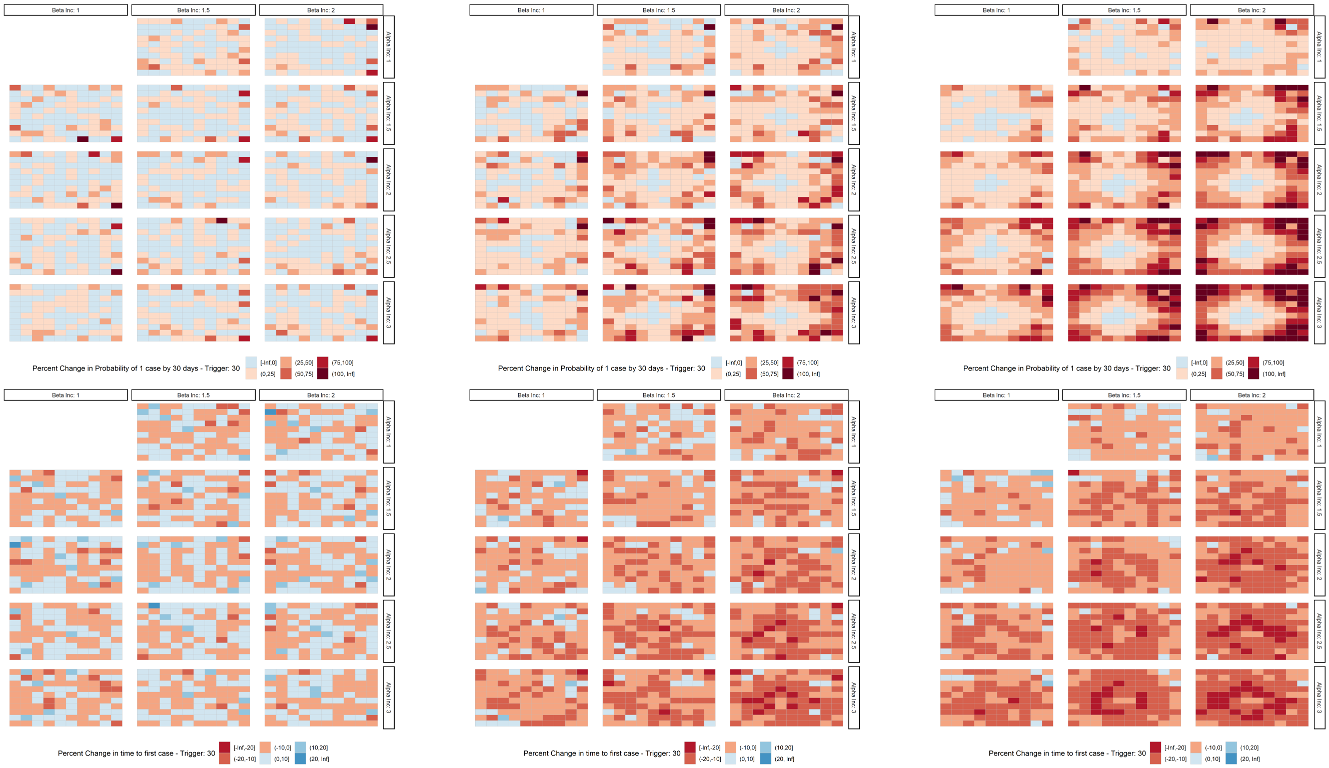

Figure S7. Top row, left to right: The percent change in the probability of having at least one case by 30 days in each location with an L_1 period (time between lockdown announcement and implementation) of 0, 3 and 7 days respectively. Bottom row, left to right: The percent change in the average number of days till the first case in each location within an L_1 period of 0, 3 and 7 days respectively.

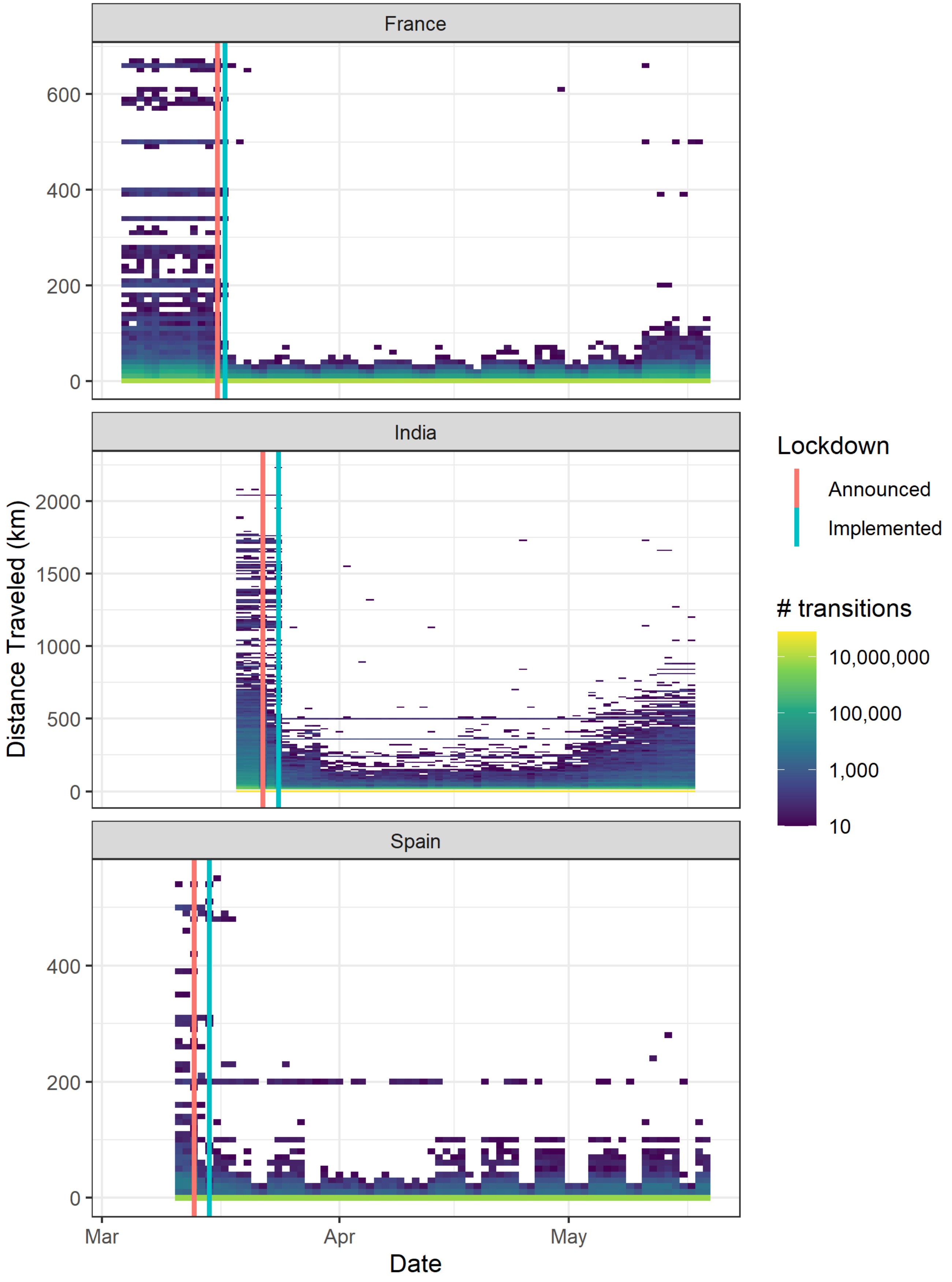

Figure S8. Number of trips made by distance. Here we can see that in all three countries, long distance trips decreased significantly in the immediate aftermath of lockdown implementation. However, local travel, including trips that ranged from 50-200 km remained during lockdown. This data is not available for Bangladesh.

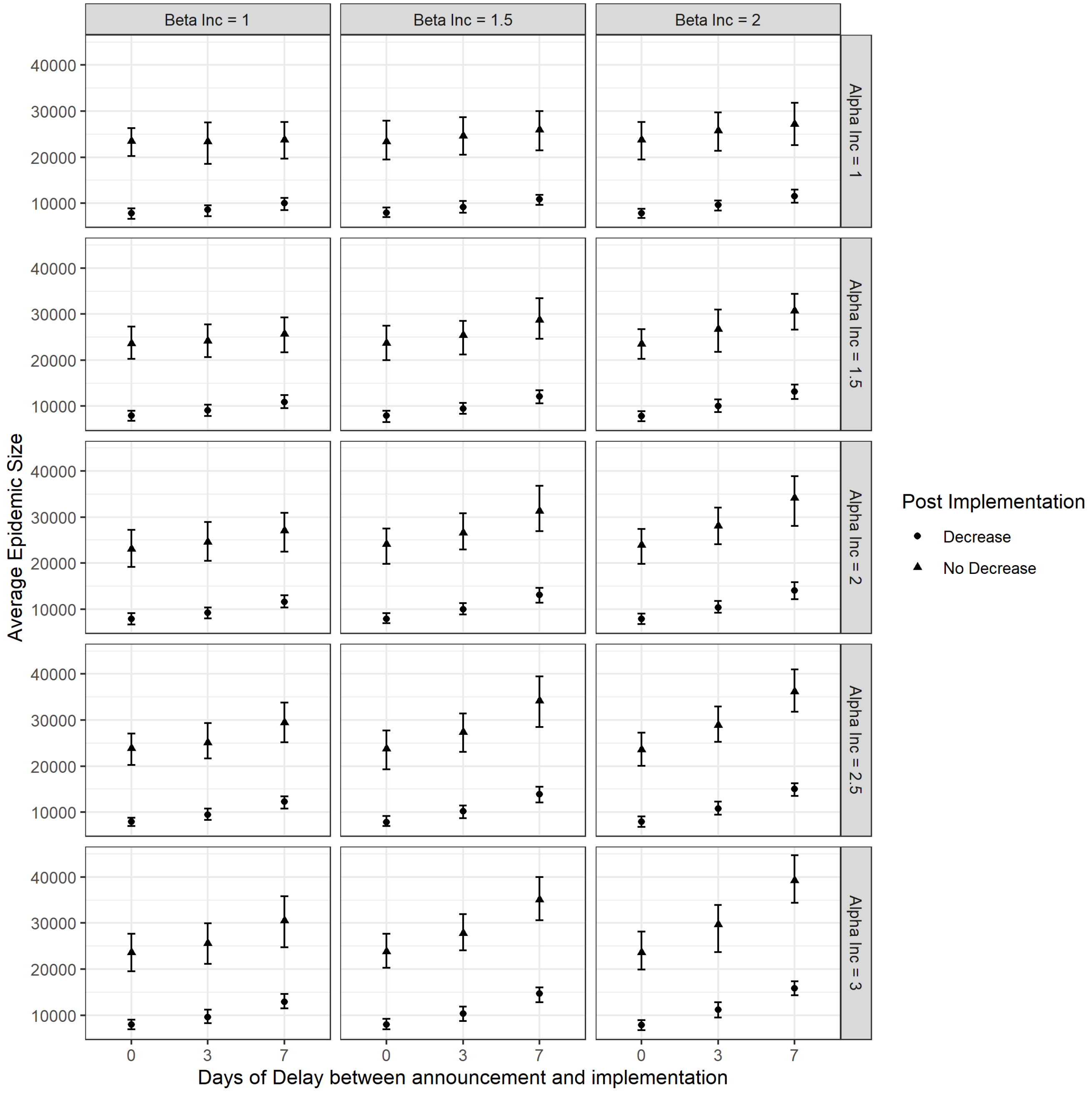

Figure S9. Overall epidemic size as over varying parameters. In all situations an epidemic with a lockdown (ie where there is a decrease in post lockdown travel) results in a smaller total epidemic size.

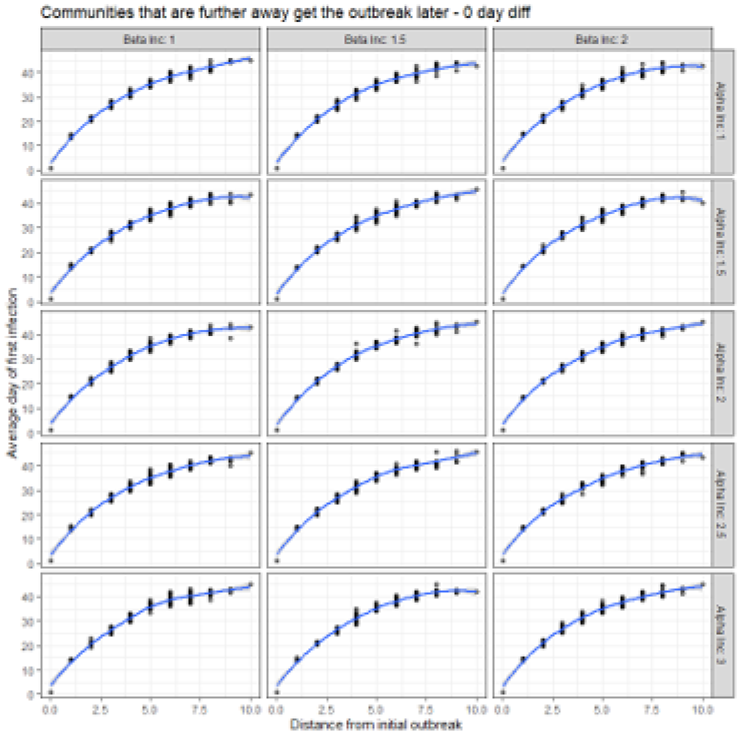

Figure S10. Average time of first case. Communities that are further away from the urban center are generally seeded later.
